# Confocal Raman Micro-Spectroscopy for Discrimination of Glycerol Diffusivity in Ex Vivo Porcine *Dura Mater*

**DOI:** 10.3390/life12101534

**Published:** 2022-10-01

**Authors:** Ali Jaafar, Maxim E. Darvin, Valery V. Tuchin, Miklós Veres

**Affiliations:** 1 Institute for Solid State Physics and Optics, Wigner Research Center for Physics, H-1525 Budapest, Hungary; 2 Institute of Physics, University of Szeged, Dom ter 9, H-6720 Szeged, Hungary; 3 Ministry of Higher Education and Scientific Research, Baghdad 10065, Iraq; 4 Center of Experimental and Applied Cutaneous Physiology, Department of Dermatology, Venerology and Allergology, Charité Universitätsmedizin Berlin, Corporate Member of Freie Universität Berlin and Humboldt-Universität zu Berlin, Charitéplatz 1, 10117 Berlin, Germany; 5 Science Medical Center, Saratov State University, 83 Astrakhanskaya Str., 410012 Saratov, Russia; 6 Laboratory of Laser Diagnostics of Technical and Living Systems, Institute of Precision Mechanics and Control, FRC “Saratov Scientific Centre of the Russian Academy of Sciences”, 24 Rabochaya Str., 410028 Saratov, Russia; 7 A.N. Bach Institute of Biochemistry, FRC “Biotechnology of the Russian Academy of Sciences”, 33-2 Leninsky Prospect, 119071 Moscow, Russia

**Keywords:** collagen type I, hydrogen bound water, high wavenumber, penetration, dehydration, glycerol, topical application, diffusion coefficients

## Abstract

**Simple Summary:**

Optical clearing is a promising method to overcome limitations in optical imaging technology for in-depth investigation. In this paper, 50% glycerol diffusivity in the framework of a passive diffusion model and water migration in ex vivo porcine *dura mater* was studied using confocal Raman micro-spectroscopy. Results show that glycerol concentration and diffusion coefficient vary at different depths, and collagen-related Raman band intensities were significantly increased for all depths after treatment. In addition, the changes in water content during optical clearing showed that 50% glycerol induces dehydration. Furthermore, these results could be translated to other fibrous biological tissues and organs.

**Abstract:**

*Dura mater* (DM) is a connective tissue with dense collagen, which is a protective membrane surrounding the human brain. The optical clearing (OC) method was used to make DM more transparent, thereby allowing to increase in-depth investigation by confocal Raman micro-spectroscopy and estimate the diffusivity of 50% glycerol and water migration. Glycerol concentration was obtained, and the diffusion coefficient was calculated, which ranged from 9.6 × 10^−6^ to 3.0 × 10^−5^ cm^2^/s. Collagen-related Raman band intensities were significantly increased for all depths from 50 to 200 µm after treatment. In addition, the changes in water content during OC showed that 50% glycerol induces tissue dehydration. Weakly and strongly bound water types were found to be most concentrated, playing a major role in the glycerol-induced water flux and OC. Results show that OC is an efficient method for controlling the DM optical properties, thereby enhancing the in-depth probing for laser therapy and diagnostics of the brain. DM is a comparable to various collagen-containing tissues and organs, such as sclera of eyes and skin dermis.

## 1. Introduction

Confocal Raman micro-spectroscopy (CRM) as an optical technique is gaining increasing attention in the medical and cosmetic research fields [1,2,3,4,5,6,7]. In the last decade, non-invasive and fast analytical methods for the real-time examination of the tissue and organ pathological and molecular modifications, diagnostics, and therapy control have become increasingly important topics. The development of optical imaging technology can play an important role in design of clinical functional cerebral probing and imaging techniques to investigate the inhomogeneity of biological objects in-depth and for laser diagnostics, therapy, and brain surgery [8,9,10]. The brain constituents, such as protein (mainly collagen), lipids, blood, and nucleic acids, can be determined using Raman spectroscopy [11,12,13]. Consequently, Raman bands provide information about the of the brain tissue conformation, when slight modification in the tissue conformation leads to change in Raman band intensities and positions, showing valuable detection and sensitivity potentials.

The Raman spectrum generally displays vibrational bands related to the substance, and the Raman bands intensity is directly proportional to concentration of the substance [14]. It gives Raman spectroscopy a promising quantification technique. Previous research has established that CRM is a non-invasive, rapid, and sensitive method to determine the penetration profiles of pro-drugs and drugs quantitatively or semi-quantitatively [15,16,17]. Various studies have related the semi-quantitative data acquired using CRM to the quantitative obtain results from alternative permeation approaches with adequate findings [18,19]. Recently, Caspers et al. [20] proposed a new method to quantify the concentration of the substances penetrated into the in vivo *stratum corneum* using CRM. Choe et al. [21] showed a huge potential of the tailored multivariate curve resolution-alternating least squares approach for calculation of the concentrations of skin components and topically applied substances. To date, no other non-invasive in vivo methods exist to quantitatively evaluate the penetration of topically applied substances into the biological objects.

*Dura mater* (DM) is the most external layer of the meninges and attached directly to the skull [22], supporting and protecting the brain in humans and animals. The meninges layers have been confirmed to act as significant aspect in nervous system physiology, traumatic brain injury, and forensic pathology. Generally, the mechanical properties of the brain have been effectively studied, and the cranial meninges layers have nearly or almost been neglected [23]. Thus, the interest of exploring the optical clearing (OC) effect on the DM’s collagen could be a suitable method to investigate human head injuries, helping the evaluation of subdural bleeding related to meninges, specifically subdural hematoma [24]. Glycerol application to the intact DM allows non-destructive visualization of DM vasculature and examination of the brain surface blood vessels using the best available methods. The advantage is to increase the ability to directly observe of human head injury (abusive head trauma) and avoid the autopsy-induced artifacts. Additionally, meningiomas are the most frequent primary tumors and are assumed to arise from DM [25,26,27]. Raman spectroscopy was effectively utilized to discriminate the differences between normal DM and tumors, correlate proteins (collagen) and lipids content [11], distinguish glioma tissue margins and different grades [28], perform intraoperative discrimination of native meningioma and DM [29], monitor the brain water content with good accuracy [30] as well as the eyes [31]. Additionally, studying the DM collagen is important to develop and improve the collagen-containing tissues, such as a graft tissue [32,33], and DM change with age [34].

All biomedical imaging and spectroscopic methods as well as CRM are suffering from crucial issues of photons limited traveling in-depth monitoring and spatial resolution due to the wavelength-dependent light scattering and absorption properties of biological tissues and organs [35]. In the case of diagnostics, imaging, and sparing therapy of brain diseases, DM is highly scattered in the range of visible and near-infrared wavelengths, restricting both the spatial resolution and probing in-depth for non-contact biomedical imaging methods [36]. Among the easiest and most efficient techniques to enhance the probing in-depth, image contrast quality, and spectroscopic information from vascular network and the cerebral cortex structures is to temporarily control/change the DM optical properties [24,37,38].

The OC technique allows for controlling/changing the optical properties of biological objects with the aim to enhance sensitivity and increase the penetration path of light photons of visible and near-infrared wavelengths into the deeper areas, which strongly enhances practical application [8,39]. The OC method is based on the administration of different optical clearing agents (OCAs) onto the biological objects, which reduces the scattering and absorption coefficients and thus enhances the imaging depth [8,40,41,42]. At present, various optical biomedical imaging methods, such as Raman spectroscopy [43], CRM [44], optical coherence tomography [45,46], 3D confocal microscopy [47], and polarized microscopy [48], are widely employed with various OCAs such as glucose [49], dimethyl sulfoxide (DMSO), glycerol [50], uDISCO [51], *ScaleS* [52], and *Scale* [53] to improve sensitivity in and increase light-photons-limited penetration into the biological objects.

Water is one of the main constituents of biological objects, comprising up to 80% of the volume. It was shown that the tissue dehydration induced by OCAs is a result of the passive diffusion of the OCAs into the biological objects, which is slower than the water migration out of the interstitial space, cells, and/or collagen fibers, and is an important mechanism of OC [54,55]. These processes offer an additional impact on the refractive index matching with OCAs due to the water content decrease in the interstitial space, containing the most portion of mobile water. These processes offer an additional impact on the refractive index matching with OCAs due to the water content decrease in the interstitial space. The application of OCAs can significantly affect the water concentration in the biological tissue [44,56]. As the hydration and water mobility states in the human skin are of great interest in cosmetology and dermatology [57,58,59], the investigation for highly effective, low-cost, non-destructive, and biocompatible OCAs with handy effect on the water concentration mobility of biological tissue for clinical use have been attracting considerable attention in recent years [60,61,62,63]. CRM was utilized in skin treated with different OCAs to investigate the changes in content of total water and water mobility states, which differ in strength of hydrogen bonds [64].

CRM is a promising technique to quantitatively monitor the efficiency of OC in real-time measurement. In this study, by using CRM, we show the results of (i) the diffusion process of 50% glycerol in ex vivo porcine DM and the diffusion coefficients calculated with the passive diffusion model; (ii) the effect of 50% glycerol on Raman intensities of the mean DM-related collagen bands; and (iii) the influence of 50% glycerol on water mobility states and total water content of ex vivo porcine DM. All these parameters are important for clinical practice due to common use of glycerol to patients undergoing neurosurgery and patients with stroke in order to decrease edema via dehydration (water flux) and thus to decrease intracranial pressure and to increase cerebral blood flow. This method could be also be used to estimate the diffusion coefficient and concentration of different OCAs in depth, such as glucose and mannitol, which are commonly use during brain surgery. DM is a comparable to various collagen tissues, such as the sclera of the eye and the dermis of the skin; therefore, the presented studies are of a rather general nature and can be applied in a wide field of medicine, including implantable smart devices for therapy [65].

## 2. Materials and Method

### 2.1. Properties of Dura Mater (DM)

The DM is a connective tissue with tough and dense collagen fibrils architecture [66], which constitutes more than 90% of DM thickness. Typical CRM probing depth for DM without OC is from 175 to 250 µm, as shown in our previous study [44]. The DM collagen fibrils’ average diameter evaluated using electron microscopy is approx. 100 ± 5 nm [67]. The refractive index for collagen fibrils and the ground matter (interstitial fluid) are 1.474 and 1.345, respectively (at 589 nm) [42]. The collagen structure of DM and, accordingly, the optical properties are similar to the sclera of the eye and the dermis of the skin. The presence of a vascular network in the DM tissue is the main contrast between the sclera of the eyes and human DM [68], which supports cranial immune cell traffic. In addition, DM is a greatly innervated tissue and thought to include the majority of meningeal lymphatic vessels [69]. It is worth noting that DM layers are anatomically and functionally connected to each other and constitute a single whole with no abrupt boundaries [67]. To study the effect of OC, fresh porcine DM was used as an appropriate in vivo human DM research model for considering different reasons such as feasibility, housing, gross anatomical structure, and ethical concerns [70,71,72].

### 2.2. OCA Reagent and DM Sample Preparation

Glycerol with ≥99.7% purity (VWR chemicals, Leuven, Belgium) was used to prepare 0 to 50% (*v*/*v*) glycerol solutions in distilled water. Then, 50% glycerol was utilized in this investigation as an OCA (refractive index: 1.415), which is safe for in vivo applications in humans [73]. In addition, glycerol has the potential to reduce intracranial pressure and brain edema [74,75,76], and the oral administration of 5% glycerol as OC enhanced imaging of in vivo brain without surgery [77].

**Figure 1:**
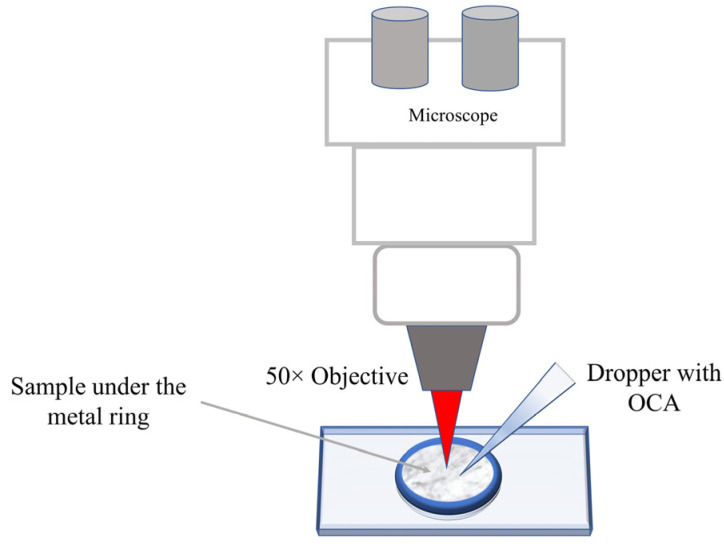
Schematic illustration of the measurement of DM samples with CRM.

Fresh porcine DM was obtained from a local slaughterhouse (Albertirsa, Hungary) and kept cold on ice in PBS. Before acquiring spectra, DM was cleaned using a paper towel, and a leather punch was utilized to cut off the DM samples of 13 mm^2^ size and 0.4 ± 0.08 mm thickness. Finally, the DM samples were placed under the metal ring for their stabilization and prevention of the glycerol from flowing downward along the lateral border of the DM surface to ensure uniform treatment (see Figure 1). The thickness of DM samples was calculated with a digital micrometer. Four samples were used to record Raman spectra in the fingerprint region for each depth and six samples in the high wavenumber region for each depth (total samples = 40 for all depths). After recording the Raman spectra, the data for each depth were averaged. All measurements were made and examined on the outermost endosteal layer.

### 2.3. Confocal Raman Micro-Spectroscopy

Raman spectra were recorded using a Renishaw inVia^TM^ confocal Raman microscope operated in backscattered geometry. The changes of 50% glycerol concentration at 50, 100, 150, and 200 μm below the DM surface with time were obtained. The measurements were performed by the focusing laser beam on the specific depth under the DM surface and then recording the spectra during the OC process. Raman spectra were recorded before and every 30 s after dropping a dose of 40 μL of 50% glycerol over the test area using a micropipette.

DM samples were held on an *xyz*-axis motorized stage with micrometer resolution computer-controlled; laser wavelength at 633 nm was used for acquiring Raman spectra with 1200 g/mm for fingerprint (FP: 400–1800 cm^−1^) and high wavenumber (HWN: 2600–3800 cm^−1^) regions and collected for 5 and 1 s acquisition time per spectrum, respectively. The measurements were performed with a spatial resolution of 0.77 µm and a laser-spot size of 1.5 µm. The CRM was calibrated using the 520 cm^−1^ Raman band position of a silicon wafer. The excitation laser light was focused on the DM surface using 50× objective, which was also used to collect Raman spectra. The laser power on the DM surface was limited to 8.3 mW, which is considered safe for the tissue. All data were obtained under the same experimental conditions at room temperature of 20 ± 1 °C.

### 2.4. Calculation of the Glycerol Concentration

**Figure 2:**
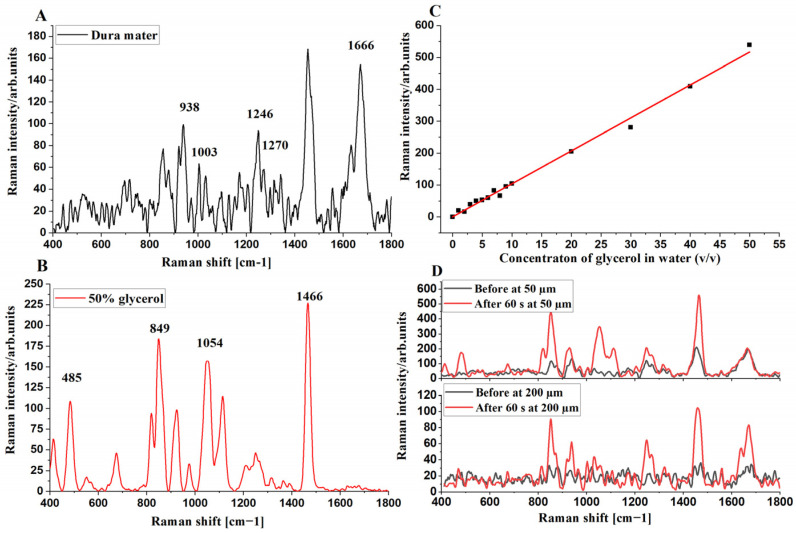
FP Raman spectra of control porcine DM at 50 µm depth (**A**), 50% glycerol (**B**), calibration curve of Raman band intensity at 485 cm^−1^, and concentration of glycerol solution in 0–50% water (**C**). The red line (in **C**) is a result of fitting with correlation coefficient (R^2^) of 0.9941 and the average Raman spectra before and after 60 s treatment at 50 and 200 µm depths (**D**).

To evaluate the glycerol concentration in DM during the OC process, the effect of the OC on the tissue optical properties and the efficiency of the Raman excitation has to be excluded. For this, first we determined the OC efficiency from the increase of the intensity of collagen-related Raman band (see Figure 2D) as OC_eff_ = *I*_OC_/*I*_0_, which is the ratio of the Raman band intensity after OCA treatment (*I*_OC_) to the Raman band intensity without treatment (*I*_0_), calculated at specific depth and time [56,78,79]. Then, we calculated the ratio of glycerol band intensities at 485 cm^−1^ measured during the experiment to OC_eff_ of different collagen-related Raman bands to obtain the change solely of the reconstruction of the glycerol concentration.

$$\mathrm{The}\;\mathrm{reconstructed}\;\mathrm{glycerol}\;\mathrm{concentration}\;\left(\mathrm{TRGC}\right)=\frac{I_{\mathrm{gly}}}{{\mathrm{OC}}_{\mathrm{eff}}}$$

Due to the OC process, there is significant crosstalk for the obtained Raman signal. The Raman signal is increased due to physical glycerol penetration and also the OC effect of the upper tissue layers; thus, we introduce the above correction approach to eliminate the influence of OC to calculate the TRGC independently of the OC efficiency from the kinetics of proteins bands because protein molecules are not movable in DM tissue.

### 2.5. Data Analysis

The Raman spectra acquired from the individual DM samples were averaged, and then the data were analyzed and processed, carried out by Spectragryph software [80]. The spectrum procedures included the baseline subtraction using a polynomial fitting and Savitzky–Golay smoothing (3rd-order polynomial and 11 points interval). Principal component analysis (PCA) was used to minimize the low-variability components of the Raman spectrum for FP and HWN regions. The PCA analysis was implemented with the built-in algorithm provided by Renishaw WIRE software package by utilizing the first three principal components. A detailed description of the PCA method can be found in the literature [81,82].

## 3. Results and Discussion

First, we recorded the Raman spectra of the control (untreated) ex vivo porcine DM at 50 µm depth, 50% glycerol, and the Raman band intensity with concentration change of glycerol solution in 0–50% water by a 633 nm wavelength (Figure 2A–C). The four collagen-related Raman bands of the DM at 938 cm^−1^ (C–C stretching mode of collagen), 1246 cm^−1^ (amide III), 1270 cm^−1^ (amide III), and 1666 cm^−1^ (amide I) [11,56,83] are clearly visible (Figure 2A).The glycerol-related Raman bands at 485, 849, 1054, and 1466 cm^−1^ are in accordance with the published results [56] and are recognized in the glycerol-treated DM (see Figure 2D) at depths 50 and 200 µm for example. Certain Raman bands of glycerol and DM are overlapped in the glycerol-treated DM (see Figure S6). Therefore, we selected the less-overlapped glycerol-related Raman band at 485 cm^−1^ to estimate the existence of glycerol in the glycerol-treated DM. In previous publications, it has been proven that glycerol applied to skin could effectively improve the Raman to fluorescence intensity ratio [56,84].

### 3.1. Calculation of the Glycerol Diffusion Coefficient and Its Effect on DM Collagen

**Figure 3:**
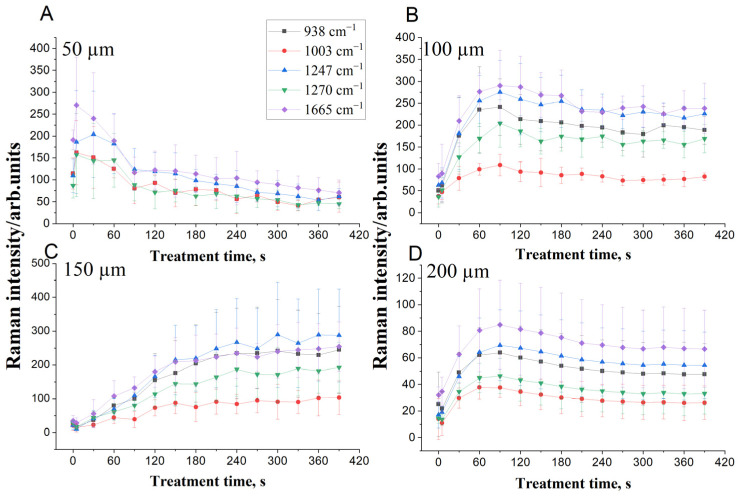
The time dependencies of DM collagen-related Raman bands at 938 (black squares), 1003 (red circles), 1247 (blue upward triangles), 1270 (green downward triangles), and 1665 cm^−1^ (purple diamonds) after impregnation by 50% aqueous-glycerol solution from 0 to 390 s at depths 50 (**A**), 100 (**B**), 150 (**C**), and 200 µm (**D**).

Figure 2C shows a linear correlation (R^2^ = 0.994) between the intensity of glycerol-related Raman band at 485 cm^−1^ and the respective concentration of glycerol in water with (total number of glycerol concentrations is 15). This calibration curve can further be utilized to calculate the TRGC in the treated DM. Following the procedure presented by Liu et al. [85], we evaluated the change in the diffusion coefficient of TRGC (*I*_gly_/OC_eff_ of collagen bands at 938, 1003, 1247, 1271, and 1665 cm^−1^) during the OC with time at different depths; for more details to explain the procedure to calculate the *C*_0_ and *D* according to these bands, see Supporting Information. At depths of 50, 100, 150, and 200 μm below the DM surface, we calculated the variation of TRGC, which is illustrated in Figures S1–S5 at 938, 1003, 1247, 1271, and 1665 cm^−1^, respectively (Supporting Information). From Figures S1–S5, we realize that the diffusion curves become smoother with increasing depth and tend towards a mono-exponential shape at 100 to 150 µm, which is agreement with the theoretical model and experimental results [85,86]. However, at 200 µm, we have at least two exponents for two competitive processes. The difference between the obtained results and theory is that we have actually two fluxes—water, which is fast, and glycerol, which is slow—and two causes for the increased glycerol Raman band intensity: one is the increase of glycerol concentration with time at a particular depth (see Figure 3C), and another is the decrease of light scattering of the DM upper layers (OC effect). Water migration is involved in the fast phase of OC, causing a dehydration effect.

To obtain the change in *D* of TRGC referring to the different probing depths of the DM, the framework of a passive diffusion model in ex vivo porcine DM samples was utilized. Considering the fibrous structures, such as the dermis of the skin, muscle, and scaler of eyes, it is totally acceptable to suppose that the dynamics of fluid diffusion within this biological object are well-expressed by free diffusion. To evaluate fluxes and in-depth glycerol concentration, according to Fick’s second law [86,87], we have an Equation (1).

(1) $$\frac{\partial C_{\mathrm{OCA}}\left(x,t\right)}{\partial t}=D\frac{\partial^{2}C_{\mathrm{OCA}}\left(x,t\right)}{\partial x^{2}}\;,$$

where *D* is the diffusion coefficient expressing the glycerol molecular transport through the porcine DM, cm^2^/s; *t* is the time after OCA is topically applied on the DM surface, s; *x* is the depth in cm. A solution for Fick’s second law for a semi-infinite medium (tissue) is shown in Equation (2).

(2) $$C_{\mathrm{OCA}}\;\left(x,t\right)=C_{0}\;\mathrm{erfc}\left(\frac{x}{2\sqrt{Dt}}\right),$$

where erfc is the complementary error function, and *C*_0_ is the highest OCA concentration at saturation conditions. For more details explaining the procedure to calculate the *C*_0_ and *D*, see Supporting Information.

For this framework model, we used non-linear curve fitting and the glycerol calibration curve (Figure 2C) to obtain the fitting parameters from Figures S1–S5, which are presented in Table S1. From Table 1, we find that the glycerol diffusion coefficient is varying at different depths, showing ascending and descending trends with time. This could be due to the tissue constituents dehydration and/or the collagen structural alteration [88]. The variation of average glycerol diffusion coefficient *D* ranged from 9.6 × 10^−6^ to 3.0 × 10^−5^ cm^2^/s depending on the depth and glycerol concentration.

The difference of the glycerol diffusion coefficients in DM depths relates to the structure and composition of DM. The DM outermost layer is the periosteal layer that adheres to the skull and contains nerves and blood vessels network, which contains elongated fibroblasts with large intercellular matrix. The middle layer is meningeal layer that contains more fibroblasts and consistently less collagen than the outermost layer [72]. Protasoni et al. [66] recognized several layers in the DM fibrous of varying structures, thicknesses, and orientation, including the bone surface, fibrous dura (external median, vascular and internal median), and arachnoid layers. From upper to in-depth, the DM collagen fibrils tend to be more chaotic and disorganized. Typically, the DM constitutes approx. 70% water, 20% collagen, and 10% elastin [89]; therefore, the diffusion coefficients of glycerol and water mixtures are a good model for study any tissue including DM. The glycerol diffusion coefficient values of mixtures of glycerol/water utilizing molecular dynamics simulation and dynamic light scattering were found to increase with decreasing glycerol concentration [90,91].

We can also recognize from Table 1 that averages of *C*_0_ range from 0.6 to 20%, which is much lower than the 50% concentration of the originally applied glycerol. The glycerol concentration decreases in depth from high to low concentration and has the highest concentration in the superficial layer. The reasons could relate to the glycerol dilution due to dehydration of tissue [92]. The fluctuation of *C*_0_ may be ascribed to the tissue micro-environment dehydration. As is can be seen in Figures S1–S5, the TRGC at 50 µm in the superficial DM significantly increases at the initial stage of the diffusion [85,86] and decreases as time passes. Nonetheless, in the deeper DM regions, the TRGC steadily increases with treatment time except for 200 µm depth. At 200 µm, the OCA concentration remains at a very small amount (Table 1, depth 200 µm); correspondingly, optical clearing efficiency is more than three times less that for other depths. However, an increase in intensity of Raman bands is enough good and fast, 1–2 min, due to low viscosity and high diffusivity of low concentrated glycerol in tissue water [90,91,93,94]. For a more prolonged observation time, saturation and even gradual decrease of intensity are seen, which can be associated with the following two phenomena. One is due to interaction of glycerol molecules with water molecules, which finally leads to binding of approximately six molecules of water by one glycerol molecule, which is the major cosmetology effect of glycerol used for tissue hydration and softening [95]. In our case, this leads to local rehydration (swelling) of tissue and its turn back to a higher scattering condition [38]. Another phenomenon is related to interaction of glycerol with collagen (protein) fibrils, which happens as a next step after filling up the interstitial space by glycerol and leads to dehydration of fibrils, at which time their refractive indices become a bit higher and surroundings a bit lower, corresponding to some refractive indices mismatching and higher light scattering [96].

The DM is extremely high-scattering tissue in the visible and near-infrared spectral regions due to the refractive indices mismatching between scatterers (collagen fibers) and the ground matter (interstitial fluid). This causes reduction of delivered laser power and distorts the focused laser, which leads to a decrease the collection of Raman signal by detectors. Therefore, the Raman signal intensities become weaker with increasing depth However, the Raman band intensities for DM treated with glycerol were found to increase in the deeper layers because glycerol improved the light propagation through the upper DM layers [44], thus permitting the studying of the deeper layers.

To study the OC effect of glycerol topically applied on the same DM samples that were used to determine the *D* of glycerol, the intensities of the five specific Raman bands were obtained in real-time measurement from 0 to 390 s at depths of 50, 100, 150, and 200 µm, respectively, below the DM surface. Figure 3 displays the kinetic curves of collagen-related Raman band intensities after glycerol treatment during OC process.

As can be seen, the Raman band intensities after the application of glycerol increased with treatment time. At 50 µm (Figure 3A), the intensity of DM bands is significantly higher during the initial stage of the glycerol treatment; it reached a maximum at 5 s and then declined due to the interaction of glycerol with mobile water in the DM for longer times. This behavior may be correlated to the time dependency of the D (see Figures S1A, S2A, S3A, S4A, and S5A) and diffusion time of glycerol caused by their complex interactions with bound and unbound water in tissue [97,98]. Another possible reason is the lack of glycerol on the surface resulting in a non-constant flux and causing the decrease of the concentration with time. The intensity of the Raman bands after a significant increase may also decrease somewhat due to local swelling of the tissue that occurs after the initial shrinkage of the tissue caused by a high concentration of glycerol (see Table 1, 50 μm), which leads to some increase in scattering, i.e., to some reduction in the efficiency of OC [38] and glycerol interaction with collagen fibrils. For the depth at 100 µm, the Raman intensity increased with treatment time and reached a maximum up to 90 s, followed by a decrease and then with saturation occurring after 210 to 390 s (Figure 3B). At the depth of 150 µm, Raman intensities monotonically and slowly increased with treatment time (Figure 3C), which can be explained by a balance between local shrinkage and swelling abilities of tissue for a particular glycerol concentration (see Table 1, 150 µm) caused by a balance of the opposite water fluxes, and thus, only relatively slow glycerol molecules diffusion provides the RI matching mechanism of OC. At the depth 200 µm, an increase in intensity of Raman bands is good and fast enough, 1–2 min, due to low viscosity and high diffusivity of low concentrated glycerol in tissue. It can be seen in Figure 3 that the Raman band intensities are increasing during OC due to the matching of refractive index and tissue dehydration caused by OCAs [99] and thus more compact collagen fibers organization and less light photons scattering. Due to a moderate glycerol concentration used in this study, we did not observe any impact associated with collagen dissociation, which is well-fitted to study of collagen dissociation by nonlinear microscopy [100]. Moreover, the OCA administration results in increased focused light penetration in depth through the DM [101,102], also leading to enhanced Raman signal intensities from deeper tissue layers.

### 3.2. OCA Effect on Water Mobility States in DM

In this section, the water mobility states were investigated in depth depending on the strength of hydrogen bonds after topical application of glycerol on six porcine DM samples ex vivo. The deconvolution-based method suggested in the literature [103,104] and adapted by Choe et al. [105] for skin was applied for in-depth analyses of DM.

To determine the water mobility states depending on the hydrogen bonds strength, the Raman spectrum of DM in the HWN region was deconvoluted using ten Gaussian functions as proposed by Choe et al. [105], and the AUC (area under the curve) of corresponding Gaussian lines was calculated. For each examined depth and treatment time, four deconvolution Gaussian functions centered at around 2850, 2880, 2940, and 2980 cm^−1^ (±5 cm^−1^) corresponded to lipids and proteins. Two Gaussian lines at 3060 and 3330 cm^−1^ (±5 cm^−1^) are related to the NH vibration of unsaturated methylene. The remaining four Gaussian lines around 3005, 3277, 3458, and 3604 cm^−1^ (±5 cm^−1^) describe the water mobility states as tightly (DAA–OH, single donor–double acceptor), strongly (DDAA–OH, double donor–double acceptor), weakly bound (DA–OH, single donor–single acceptor), and free water (sum of very weakly bound (DDA–OH, double donor–single acceptor) and unbound water (free OH)), as proposed in [105]. The widths of Gaussian lines obtained were authorized for modification within 20 cm^−1^ for better-fitting quality at different depths of DM and treatment time. The full water concentration was determined as a ratio of the sum of the AUCs of all four water states to the concentration of protein (AUC of the Gaussian line function at ≈2940 cm^−1^).

**Figure 4:**
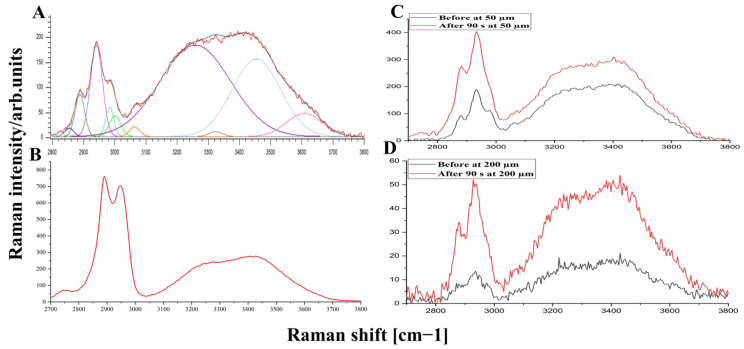
Gaussian lines function-based deconvolution of the HWN Raman spectrum of untreated porcine DM at 50 μm (**A**), 50% glycerol water solution (**B**), and the average of Raman spectra before and after 90 s treatment at 50 and 200 μm depths (**C** and **D**), respectively.

Figure 4 displays the deconvolution-based procedure applied to Raman spectrum of untreated DM (Figure 4A), Raman spectrum of 50% glycerol in water solution (Figure 4B), and the effect of OC on depths at 50 and 200 µm (Figure 4C,D), respectively, in the HWN region. It can clearly be seen that 50% glycerol in water solution has broad 2910 to 2965 cm^−1^ (CH_2_ vibrations of OCA) and 3100 to 3700 cm^−1^ (CO–H and OH vibrations) Raman bands [106], which are overlapped with Raman bands of protein and water in the DM tissue. Therefore, glycerol could have an influence on the obtained results.

**Figure 5:**
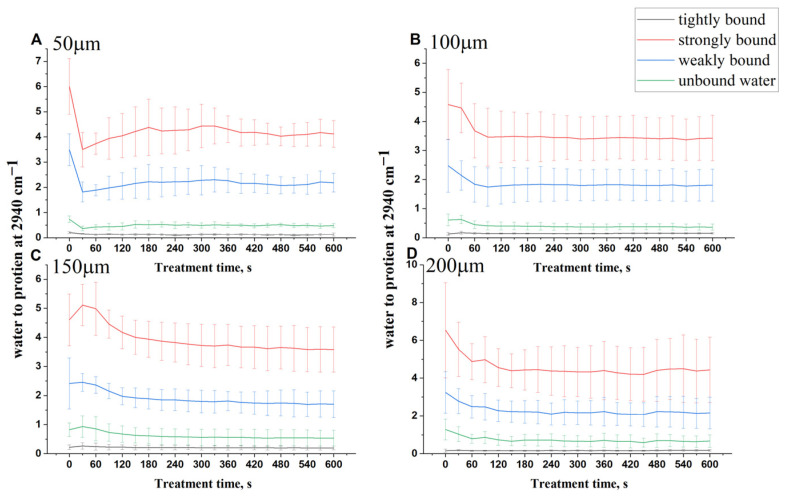
The time-dependent kinetics of 3005 cm^−1^ (tightly bound water, black), 3270 cm^−1^ (strongly bound water, red), 3458 cm^−1^ (weakly bound water, blue), and 3605 cm^−1^ (unbound water, green) deconvoluted Gaussian lines at depths of 50 (**A**), 100 (**B**), 150 (**C**), and 200 µm (**D**).

To study the influence of glycerol on the water mobility states in the DM, the AUC values were calculated for tightly, strongly, and weakly bound and un-bound water molecule states with time at different depths. In addition, all values were normalized to the AUC of the protein-related Gaussian line at 2940 cm^−1^. The results are illustrated in Figure 5.

Figure 5 shows the treatment time-dependent kinetics of the four different water mobility states for DM treated with 50% glycerol solution. As can be seen from Figure 5, all water states decreased at all depths with respect to time after application of glycerol except the tightly bound water concentration, which has the fewest changes after treatment in all depths. At a depth 50 µm of glycerol-treated DM (Figure 5A), strongly bound and weakly bound water molecules were significantly reduced at the beginning and then slightly increased after 60 s. This can be associated with the rehydration of collagen in DM due to the water replacement in the upper tissue depth with water accumulated from deeper tissue layers [107]. At depths of 100, 150, and 200 µm, the strongly bound and weakly bound water monotonically decrease with time. Moreover, it can be seen that the unbound water decreased with treatment time for all four depths (Figure 5B–D).

Table 2 shows the reduction in the concentration of four water mobility states after application of glycerol, namely tightly bound (3005 cm^−1^), strongly bound (2370 cm^−1^), weakly bound (3458 cm^−1^), and unbound (3605 cm^−1^) water at the depths of 50, 100, 150, and 200 µm.

Furthermore, it is noticeable from Table 2 that the highest-concentrated water molecule states at all probed depths of DM are strongly bound and weakly bound water molecule states. It is obvious that the values related to these two types are significantly reduced at all probing depths after 50% glycerol treatment. The obtained results display that in the DM, strongly and weakly bound water are preferentially involved in the OCA-induced water flux and thus have a major contribution in the OC. Similar behavior is observed in the skin [64,105].

**Figure 6:**
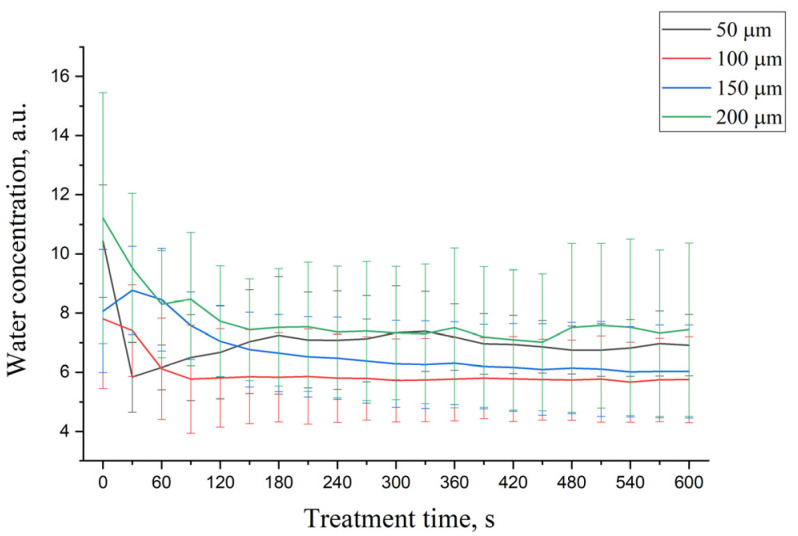
The kinetics of total water content changes in 50% glycerol-treated porcine DM at depths: 50 (black), 100 (red), 150 (blue), and 200 µm (green) depending on treatment time.

Figure 6 shows the influence of glycerol on the total water content in DM. As illustrated in Figure 6, the total water molecule content in DM decreases in time for all depths except at 50 µm, where it rapidly decreases at the beginning of treatment time followed by an increase with treatment time due to the water replacement in the upper tissue depth with water accumulated from deeper tissue layers [107].

However, the obtained results can be potentially underestimated [108], as 50% glycerol has its own Raman band in the 2910–2965 cm^−1^ region, where DM has a protein-related Raman band. Nevertheless, this underestimation is more pronounced in the superficial depth, where the glycerol has maximum concentration. In deeper DM layers, where the glycerol exists in much lower concentrations, it can be assumed that the contribution from the glycerol-related Raman band is significantly less compared to the protein-related band of the DM, thus substantially reducing the underestimation. It is crucial to highlight that the amplified osmotic pressure induced by glycerol treatment results in the transition of bound into unbound water states [98,109], which is similar to the effect observed during occlusion-induced skin swelling [110]. Thus, glycerol can induce local depth-dependent dehydration of DM. In addition, when the glycerol enters the DM, glycerol molecules are bound with water molecules of tissue. This leads to the occurrence of fluxes water content between glycerol-bound water and DM’s water.

## 4. Conclusions

Confocal Raman micro-spectroscopy proved to be a rapid and highly sensitive technique to determine the glycerol diffusion coefficients in DM. Our results confirm that the diffusion coefficients of glycerol in ex vivo porcine DM vary at various depths ranging from 9.6 × 10^−6^ to 3.0 × 10^−5^ cm^2^/s. The highest glycerol concentration (*C*_0_) reached in the DM is about 20%, which is much less than the originally used concentration of 50%. The DM-related Raman band intensities significantly increased after treatment with 50% glycerol solution for all depths, thus confirming the OC effect. It also shows that the application of glycerol results in significant changes in DM total water profile. As most prevalent water states in the DM are weakly and strongly bound water, they favorably participate in the water flux in the OCA-treated DM and therefore have a major contribution to the OC. The results are valid for various laser therapy, optical biomedical diagnostics, and imaging methods. For instance, in laser therapy of the brain vascular network, the high attenuation and broadening of the laser beam by tissue scattering is frequently a challenge. OCAs will permit a laser beam to be directly focused on a blood vessel network. A smaller spot size of the laser beam will reduce the laser-induced collateral damage of the tissue. OCs reduce the scattering of the surrounding tissue impregnated by the OCAs, vascular issues, cancers, and other inhomogeneities in tissue that can be imaged at higher resolutions. Furthermore, these results could be applied to many other fibrous biological tissues. Additionally, these obtained results could be valuable for forensic pathology, Burr hole evacuation of intracranial subdural hematomas, and traumatic brain injury study, as well as for optical monitoring of smart implants.

## Figures and Tables

**Table 1:** The average of glycerol diffusion coefficient and concentration of the fitting data from the Table S1.

	Depth 50 µm		Depth 150 µm	
**Raman bands**	***D* (cm^2^/s)**	***C*_0_**	***D* (cm^2^/s)**	***C*_0_**
938 cm^−1^	3.0 × 10^−6^	21	9.4 × 10^−6^	1.2
1003 cm^−1^	2.5 × 10^−6^	27	6.5 × 10^−5^	2.3
1247 cm^−1^	3.5 × 10^−5^	11	2.2 × 10^−5^	1.1
1270 cm^−1^	5.0 × 10^−6^	15	9.6 × 10^−6^	1.9
1665 cm^−1^	2.3 × 10^−6^	26	4.2 × 10^−6^	1.7
Average	9.6 × 10^−6^	20	2.2 × 10^−5^	1.6
	**Depth 100 µm**		**Depth 200 µm**	
**Raman bands**	***D* (cm^2^/s)**	***C*_0_**	***D* (cm^2^/s)**	***C*_0_**
938 cm^−1^	1.7 × 10^−6^	3.4	9.8 × 10^−5^	0.6
1003 cm^−1^	2.7 × 10^−6^	4.2	6.2 × 10^−6^	0.8
1247 cm^−1^	5.9 × 10^−6^	2.4	8.1 × 10^−6^	0.5
1270 cm^−1^	4.6 × 10^−5^	1.5	1.1 × 10^−5^	0.6
1665 cm^−1^	4.3 × 10^−6^	3.1	2.5 × 10^−5^	0.6
Average	1.2 × 10^−5^	2.9	3.0 × 10^−5^	0.6

**Table 2:** The change of four water mobility states before and after the treatment of porcine DM with 50% glycerol in water solution.

Depths	50 µm		100 µm		150 µm		200 µm	
Water Types	Before	After	Before	After	Before	After	Before	After
Tightly bound	0.20	0.12	0.13	0.15	0.22	0.19	0.18	0.177
Strongly bound	6.00	4.12	4.57	3.42	4.60	3.58	6.53	4.44
Weakly bound	3.49	2.18	2.47	1.80	2.41	1.70	3.24	2.15
Unbound water	0.73	0.48	0.61	0.36	0.82	0.53	1.27	0.66

## Data Availability

The data presented in this study are available on request from the corresponding author.

## Supplementary Materials

The following supporting information can be downloaded at: https://www.mdpi.com/article/10.3390/life12101534/s1, Figures S1–S5: The change of TRGC with treatment time at different depths of 50 (A), 100 (B), 150 (C), and 200 μm (D) for porcine DM samples at 938, 1003, 1247, 1270, and 1665 cm^−1^, resulting from the fitting data using the passive diffusion model; Figure S6: Specific region of FP Raman spectra of control porcine DM at 50 µm depth (red) and 50% glycerol (black) where the overlap of the protein-related DM Raman bands at 938, 1248, and 1270 cm^−1^ are minimal; Table S1: Results of the fitting data of the Figures S1–S5 for DM collagen at different depths and treatment times using the passive diffusion model. Reference [111] is cited in the supplementary materials.

## Abbreviations

CRM, confocal Raman micro-spectroscopy; DM, *dura mater*; OCAs, optical clearing agents; OC, optical clearing; *D*, diffusion coefficient; TRGC, the reconstructed glycerol concentration.

## References

[1] Latka I., Dochow S., Krafft C., Dietzek B., Popp J. (2013). Fiber optic probes for linear and nonlinear Raman applications-Current trends and future development. Laser Photonics Rev..

[2] Darvin M.E., Schleusener J., Lademann J., Choe C.-S. (2022). Current views on non-invasive in vivo determination of physiological parameters of the stratum corneum using confocal Raman microspectroscopy. Skin Pharmacol. Physiol..

[3] DePaoli D., Lemoine É., Ember K., Parent M., Prud’homme M., Cantin L., Petrecca K., Leblond F., Côté D.C. (2020). Rise of Raman spectroscopy in neurosurgery: A review. J. Biomed. Opt..

[4] Stevens A.R., Stickland C.A., Harris G., Ahmed Z., Oppenheimer P.G., Belli A., Davies D.J. (2022). Raman Spectroscopy as a Neuromonitoring Tool in Traumatic Brain Injury: A Systematic Review and Clinical Perspectives. Cells.

[5] Lee K.S., Landry Z., Pereira F.C., Wagner M., Berry D., Huang W.E., Taylor G.T., Kneipp J., Popp J., Zhang M. (2021). Raman microspectroscopy for microbiology. Nat. Rev. Methods Prim..

[6] Azemtsop Matanfack G., Pistiki A., Rösch P., Popp J. (2021). Raman stable isotope probing of bacteria in visible and deep uv-ranges. Life.

[7] Mandrell C.T., Holland T.E., Wheeler J.F., Esmaeili S.M.A., Amar K., Chowdhury F., Sivakumar P. (2020). Machine learning approach to raman spectrum analysis of mia paca-2 pancreatic cancer tumor repopulating cells for classification and feature analysis. Life.

[8] Tuchin V.V. (2015). Tissue Optics: Light Scattering Methods and Instruments for Medical Diagnostics.

[9] Tuchin V.V. (2016). Handbook of Optical Biomedical Diagnostics. Light-Tissue Interaction.

[10] Neprokin A., Broadway C., Myllylä T., Bykov A., Meglinski I. (2022). Photoacoustic Imaging in Biomedicine and Life Sciences. Life.

[11] Koljenović S., Schut T.B., Vincent A., Kros J.M., Puppels G.J. (2005). Detection of meningioma in dura mater by Raman spectroscopy. Anal. Chem..

[12] Mizuno A., Hayashi T., Tashibu K., Maraishi S., Kawauchi K., Ozaki Y. (1992). Near-infrared FT-Raman spectra of the rat brain tissues. Neurosci. Lett..

[13] Mizuno A., Kitajima H., Kawauchi K., Muraishi S., Ozaki Y. (1994). Near-infrared Fourier transform Raman spectroscopic study of human brain tissues and tumours. J. Raman Spectrosc..

[14] Aarnoutse P.J., Westerhuis J.A. (2005). Quantitative Raman reaction monitoring using the solvent as internal standard. Anal. Chem..

[15] Tfayli A., Piot O., Pitre F., Manfait M. (2007). Follow-up of drug permeation through excised human skin with confocal Raman microspectroscopy. Eur. Biophys. J..

[16] Tippavajhala V.K., de Oliveira Mendes T., Martin A.A. (2018). In Vivo Human Skin Penetration Study of Sunscreens by Confocal Raman Spectroscopy. AAPS Pharm. Sci. Tech..

[17] Tfaili S., Josse G., Angiboust J.F., Manfait M., Piot O. (2014). Monitoring caffeine and resveratrol cutaneous permeation by confocal Raman microspectroscopy. J. Biophotonics.

[18] Mohammed D., Matts P.J., Hadgraft J., Lane M.E. (2014). In vitro-in vivo correlation in skin permeation. Pharm. Res..

[19] Mateus R., Abdalghafor H., Oliveira G., Hadgraft J., Lane M.E. (2013). A new paradigm in dermatopharmacokinetics-Confocal Raman spectroscopy. Int. J. Pharm..

[20] Caspers P.J., Nico C., Bakker Schut T.C., Sterke J., Pudney P.D.A., Curto P.R., Illand A., Puppels G.J. (2019). Method to quantify the in vivo skin penetration of topically applied materials based on confocal Raman spectroscopy. Transl. Biophotonics.

[21] Choe C.S., Ri J.S., Choe S.H., Kim P.S., Lademann J., Schleusener J., Darvin M.E. (2022). tMCR-ALS method for the determination of water concentration profiles in the stratum corneum of untreated and treated skin in vivo. J. Raman Spectrosc..

[22] Jacobson S., Marcus E.M., Stanley P. (2018). Neuroanatomy for the Neuroscientist.

[23] Walsh D.R., Ross A.M., Newport D.T., Zhou Z., Kearns J., Fearon C., Lorigan J., Mulvihill J.J.E. (2021). Mechanical characterisation of the human dura mater, falx cerebri and superior sagittal sinus. Acta Biomater..

[24] Cheshire E.C., Malcomson R.D.G., Joseph S., Biggs M.J.B., Adlam D., Rutty G.N. (2015). Optical clearing of the dura mater using glycerol: A reversible process to aid the post-mortem investigation of infant head injury. Forensic. Sci. Med. Pathol..

[25] Riemenschneider M.J., Perry A., Reifenberger G. (2006). Histological classification and molecular genetics of meningiomas. Lancet Neurol..

[26] Umana G.E., Scalia G., Vats A., Pompili G., Barone F., Passanisi M., Graziano F., Maugeri R., Tranchina M.G., Cosentino S. (2021). Primary extracranial meningiomas of the head and neck. Life.

[27] Alam S., Ferini G., Muhammad N., Ahmed N., Wakil A.N.M., Islam K.M.A., Arifin M.S., Al Mahbub A., Habib R., Mojumder M.R. (2022). Skull Base Approaches for Tuberculum Sellae Meningiomas: Institutional Experience in a Series of 34 Patients. Life.

[28] Zhou Y., Liu C.-H., Wu B., Yu X., Cheng G., Zhu K., Wang K., Zhang C., Zhao M., Zong R. (2019). Optical biopsy identification and grading of gliomas using label-free visible resonance Raman spectroscopy. J. Biomed. Opt..

[29] Jelke F., Mirizzi G., Borgmann F.K., Husch A., Slimani R., Klamminger G.G., Klein K., Mombaerts L., Gérardy J.J., Mittelbronn M. (2021). Intraoperative discrimination of native meningioma and dura mater by Raman spectroscopy. Sci. Rep..

[30] Wolthuis R., van Aken M., Fountas K., Robinson J.S., Bruining H.A., Puppels G.J. (2001). Determination of water concentration in brain tissue by Raman spectroscopy. Anal. Chem..

[31] Huizinga A., Bot A.C.C., de Mul F.F.M., Vrensen G.F.J.M., Greve J. (1989). Local variation in absolute water content of human and rabbit eye lenses measured by Raman microspectroscopy. Exp. Eye Res..

[32] Calikoglu C., Cakir M., Tuzun Y. (2019). Histopathological investigation of the effectiveness of collagen matrix in the repair of experimental spinal dura mater defects. Eurasian J. Med..

[33] Liu W., Wang X., Su J., Jiang Q., Wang J., Xu Y., Zheng Y., Zhong Z., Lin H. (2021). In vivo Evaluation of Fibrous Collagen Dura Substitutes. Front. Bioeng. Biotechnol..

[34] Zwirner J., Scholze M., Waddell J.N., Ondruschka B., Hammer N. (2019). Mechanical Properties of Human Dura Mater in Tension–An Analysis at an Age Range of 2 to 94 Years. Sci. Rep..

[35] Zhang Y., Liu H., Tang J., Li Z., Zhou X., Zhang R., Chen L., Mao Y., Li C. (2017). Noninvasively Imaging Subcutaneous Tumor Xenograft by a Handheld Raman Detector, with the Assistance of an Optical Clearing Agent. ACS Appl. Mater. Interfaces.

[36] Bashkatov A.N., Genina E.A., Kochubey V.I., Sinichkin Y.P., Korobov A.A., Lakodina N.A., Tuchin V.V. (2000). In vitro study of control of human dura mater optical properties by acting of osmotical liquids. Control. Tissue Opt. Prop. Appl. Clin. Study.

[37] Genina E.A., Bashkatov A.N., Kochubey V.I., Tuchin V.V. (2005). Optical clearing of human dura mater. Opt. Spectrosc..

[38] Genina E., Bashkatov A., Tuchin V. (2017). Optical clearing of human dura mater by glucose solutions. J. Biomed. Photonics Eng..

[39] Tuchin V.V., Maksimova I.L., Zimnyakov D.A., Kon I.L., Mavlutov A.K., Mishin A.A. (1997). Light propagation in tissues with controlled optical properties. J. Biomed. Opt..

[40] Sdobnov A., Darvin M., Genina E., Bashkatov A., Lademann J., Tuchin V. (2018). Recent progress in tissue optical clearing for spectroscopic application. Spectrochim. Acta Part A Mol. Biomol. Spectrosc..

[41] Oliveira L.M.C., Tuchin V.V. (2019). The Optical Clearing Method-A New Tool for Clinical Practice and Biomedical Engineering.

[42] Tuchin V.V. (2005). Optical Clearing of Tissues and Blood.

[43] Schulmerich M.V., Cole J.H., Dooley K.A., Morris M.D., Kreider J.M., Goldstein S.A. (2008). Optical clearing in transcutaneous Raman spectroscopy of murine cortical bone tissue. J. Biomed. Opt..

[44] Jaafar A., Holomb R., Sdobnov A.Y., Ocskay Z., Jakus Z., Tuchin V.V., Veres M. (2022). Ex vivo confocal Raman microspectroscopy of porcine dura mater supported by optical clearing. J. Biophotonics.

[45] Zhernovaya O., Tuchin V.V., Leahy M.J. (2016). Enhancement of OCT imaging by blood optical clearing in vessels-A feasibility study. Photonics Lasers Med..

[46] Liang Y., Yuan W., Mavadia-Shukla J., Li X. (2016). Optical clearing for luminal organ imaging with ultrahigh-resolution optical coherence tomography. J. Biomed. Opt..

[47] Fu Y.Y., Tang S.C. (2010). Optical clearing facilitates integrated 3D visualization of mouse ileal microstructure and vascular network with high definition. Microvasc. Res..

[48] Nadiarnykh O., Campagnola P.J. (2009). Retention of polarization signatures in SHG microscopy of scattering tissues through optical clearing. Opt. Express.

[49] Larin K.V., Tuchin V.V. (2008). Functional imaging and assessment of the glucose diffusion rate in epithelial tissues in optical coherence tomography. Quantum Electron..

[50] Zhu X., Huang L., Zheng Y., Song Y., Xu Q., Wang J., Si K., Duan S., Gong W. (2019). Ultrafast optical clearing method for three-dimensional imaging with cellular resolution. Proc. Natl. Acad. Sci. USA.

[51] Pan C., Cai R., Quacquarelli F.P., Ghasemigharagoz A., Lourbopoulos A., Matryba P., Plesnila N., Dichgans M., Hellal F., Ertürk A. (2016). Shrinkage-mediated imaging of entire organs and organisms using uDISCO. Nat. Methods.

[52] Hama H., Hioki H., Namiki K., Hoshida T., Kurokawa H., Ishidate F., Kaneko T., Akagi T., Saito T., Saido T. (2015). ScaleS: An optical clearing palette for biological imaging. Nat. Neurosci..

[53] Hama H., Kurokawa H., Kawano H., Ando R., Shimogori T., Noda H., Fukami K., Sakaue-Sawano A., Miyawaki A. (2011). Scale: A chemical approach for fluorescence imaging and reconstruction of transparent mouse brain. Nat. Neurosci..

[54] Tuchin V.V., Zhu D., Genina E.A. (2022). Handbook of Tissue Optical Clearing New Prospects in Optical Imaging Edited.

[55] Guo X., Guo Z.Y., Wei H.J., Yang H.Q., He Y.H., Xie S.S., Wu G.Y., Zhong H.Q., Li L.Q., Zhao Q.L. (2010). In vivo quantification of propylene glycol, glucose and glycerol diffusion in human skin with optical coherence tomography. Laser Phys..

[56] Sdobnov A.Y., Tuchin V.V., Lademann J., Darvin M.E. (2017). Confocal Raman microscopy supported by optical clearing treatment of the skin-Influence on collagen hydration. J. Phys. D Appl. Phys..

[57] Egawa M., Yanai M., Maruyama N., Fukaya Y., Hirao T. (2015). Visualization of water distribution in the facial epidermal layers of skin using high-sensitivity near-infrared (NIR) imaging. Appl. Spectrosc..

[58] Boireau-Adamezyk E., Baillet-Guffroy A., Stamatas G.N. (2014). Mobility of water molecules in the stratum corneum: Effects of age and chronic exposure to the environment. J. Investig. Dermatol..

[59] Akdeniz M., Boeing H., Müller-Werdan U., Aykac V., Steffen A., Schell M., Blume-Peytavi U., Kottner J. (2018). Effect of Fluid Intake on Hydration Status and Skin Barrier Characteristics in Geriatric Patients: An Explorative Study. Skin Pharmacol. Physiol..

[60] Oliveira L.M., Carvalho M.I., Nogueira E.M., Tuchin V.V. (2018). Skeletal muscle dispersion (400–1000 nm) and kinetics at optical clearing. J. Biophotonics.

[61] Carneiro I., Carvalho S., Henrique R., Oliveira L., Tuchin V.V. (2017). Simple multimodal optical technique for evaluation of free/bound water and dispersion of human liver tissue. J. Biomed. Opt..

[62] Shi R., Guo L., Zhang C., Feng W., Li P., Ding Z., Zhu D. (2017). A useful way to develop effective in vivo skin optical clearing agents. J. Biophotonics.

[63] Behm P., Hashemi M., Hoppe S., Wessel S., Hagens R., Jaspers S., Wenck H., Rübhausen M. (2017). Confocal spectroscopic imaging measurements of depth dependent hydration dynamics in human skin in-vivo. AIP Adv..

[64] Sdobnov A.Y., Darvin M.E., Schleusener J., Lademann J., Tuchin V.V. (2019). Hydrogen bound water profiles in the skin influenced by optical clearing molecular agents—Quantitative analysis using confocal Raman microscopy. J. Biophotonics.

[65] Lee J.S., Kim J., Ye Y.S., Kim T.I. (2022). Materials and device design for advanced phototherapy systems. Adv. Drug Deliv. Rev..

[66] Protasoni M., Sangiorgi S., Cividini A., Culuvaris G.T., Tomei G., Dell’Orbo C., Raspanti M., Balbi S., Reguzzoni M. (2011). The collagenic architecture of human dura mater: Laboratory investigation. J. Neurosurg..

[67] Bashkatov A.N., Genina E.A., Sinichkin Y.P., Kochubey V.I., Lakodina N.A., Tuchin V.V. (2003). Glucose and Mannitol Diffusion in Human Dura Mater. Biophys. J..

[68] Rua R., McGavern D.B. (2018). Advances in Meningeal Immunity. Trends Mol. Med..

[69] Absinta M., Ha S.-K., Nair G., Sati P., Luciano N.J., Palisoc M., Louveau A., Zaghloul K.A., Pittaluga S., Kipnis J. (2017). Human and nonhuman primate meninges harbor lymphatic vessels that can be visualized noninvasively by MRI. Elife.

[70] Frink M., Andruszkow H., Zeckey C., Krettek C., Hildebrand F. (2011). Experimental trauma models: An update. J. Biomed. Biotechnol..

[71] Mazgajczyk E., Ścigała K., Czyż M., Jarmundowicz W., Będziński R. (2012). Mechanical properties of cervical dura mater. Acta Bioeng. Biomech..

[72] Kinaci A., Bergmann W., Bleys R.L.A.W., van der Zwan A., van Doormaal T.P.C. (2020). Histologic comparison of the dura mater among species. Comp. Med..

[73] Lai J.H., Liao E.Y., Liao Y.H., Sun C.K. (2021). Investigating the optical clearing effects of 50% glycerol in ex vivo human skin by harmonic generation microscopy. Sci. Rep..

[74] Chang C.Y., Pan P.H., Li J.R., Ou Y.C., Liao S.L., Chen W.Y., Kuan Y.H., Chen C.J. (2021). Glycerol improves intracerebral hemorrhagic brain injury and associated kidney dysfunction in rats. Antioxidants.

[75] Berger C., Sakowitz O.W., Kiening K.L., Schwab S. (2005). Neurochemical monitoring of glycerol therapy in patients with ischemic brain edema. Stroke.

[76] Sakamaki M., Igarashi H., Nishiyama Y., Hagiwara H., Ando J., Chishiki T., Curran B.C., Katayama Y. (2003). Effect of glycerol on ischemic cerebral edema assessed by magnetic resonance imaging. J. Neurol. Sci..

[77] Iijima K., Oshima T., Kawakami R., Nemoto T. (2021). Optical clearing of living brains with MAGICAL to extend in vivo imaging. iScience.

[78] Jaafar A., Mahmood M.H., Holomb R., Himics L., Váczi T., Sdobnov A.Y., Tuchin V.V., Veres M. (2021). Ex-vivo confocal Raman microspectroscopy of porcine skin with 633/785-NM laser excitation and optical clearing with glycerol/water/DMSO solution. J. Innov. Opt. Health Sci..

[79] Yanina I.Y., Schleusener J., Lademann J., Tuchin V.V., Darvin M.E. (2020). The Effectiveness of Glycerol Solutions for Optical Clearing of the Intact Skin as Measured by Confocal Raman Microspectroscopy. Opt. Spectrosc..

[80] Spectragryph-Optical Spectroscopy Software: Description, (n.d.). https://www.effemm2.de/spectragryph/about_descr.html.

[81] Mujica Ascencio S., Choe C.S., Meinke M.C., Müller R.H., Maksimov G.V., Wigger-Alberti W., Lademann J., Darvin M.E. (2016). Confocal Raman microscopy and multivariate statistical analysis for determination of different penetration abilities of caffeine and propylene glycol applied simultaneously in a mixture on porcine skin ex vivo. Eur. J. Pharm. Biopharm..

[82] Choe C., Lademann J., Darvin M.E. (2016). A depth-dependent profile of the lipid conformation and lateral packing order of the stratum corneum in vivo measured using Raman microscopy. Analyst.

[83] Pezzotti G., Boffelli M., Miyamori D., Uemura T., Marunaka Y., Zhu W., Ikegaya H. (2015). Raman spectroscopy of human skin: Looking for a quantitative algorithm to reliably estimate human age. J. Biomed. Opt..

[84] Huang D., Zhang W., Zhong H., Xiong H., Guo X., Guo Z. (2012). Optical clearing of porcine skin tissue in vitro studied by Raman microspectroscopy. J. Biomed. Opt..

[85] Liu P., Huang Y., Guo Z., Wang J., Zhuang Z., Liu S. (2013). Discrimination of dimethyl sulphoxide diffusion coefficient in the process of optical clearing by confocal micro-Raman spectroscopy. J. Biomed. Opt..

[86] Zhou F., Wang R.K. (2004). Theoretical model on optical clearing of biological tissue with semipermeable chemical agents. Complex Dyn. Fluct. Chaos Fractals Biomed. Photonics.

[87] Bashkatov A.N., Tuchin V.V., Genina E.A., Stolnitz M.M., Zhestkov D.M., Altshuler G.B., Yaroslavsky I.V. (2007). Monte Carlo study of skin optical clearing to enhance light penetration in the tissue. Complex Dyn. Fluct. Biomed. Photonics IV.

[88] Zhuo S., Chen J., Wu G., Xie S., Zheng L., Jiang X., Zhu X. (2010). Quantitatively linking collagen alteration and epithelial tumor progression by second harmonic generation microscopy. Appl. Phys. Lett..

[89] Takeuchi M., Hayakawa S., Ichikawa A., Hasegawa A., Hasegawa Y., Fukuda T. (2020). Multilayered artificial dura-mater models for a minimally invasive brain surgery simulator. Appl. Sci..

[90] Akinkunmi F.O., Jahn D.A., Giovambattista N. (2015). Effects of temperature on the thermodynamic and dynamical properties of glycerol-water mixtures: A computer simulation study of three different force fields. J. Phys. Chem. B.

[91] Rausch M.H., Heller A., Fröba A.P. (2017). Binary Diffusion Coefficients of Glycerol-Water Mixtures for Temperatures from 323 to 448 K by Dynamic Light Scattering. J. Chem. Eng. Data.

[92] Rylander C.G., Stumpp O.F., Milner T.E., Kemp N.J., Mendenhall J.M., Diller K.R., Welch A.J. (2006). Dehydration mechanism of optical clearing in tissue. J. Biomed. Opt..

[93] D’Errico G., Ortona O., Capuano F., Vitagliano V. (2004). Diffusion coefficients for the binary system glycerol + water at 25 °C. A velocity correlation study. J. Chem. Eng. Data.

[94] Egorov A.V., Lyubartsev A.P., Laaksonen A. (2011). Molecular dynamics simulation study of glycerol-water liquid mixtures. J. Phys. Chem. B.

[95] Fluhr J.W., Darlenski R., Surber C. (2008). Glycerol and the skin: Holistic approach to its origin and functions. Br. J. Dermatol..

[96] Tuchin V.V. (1999). Coherent Optical Techniques for The Analysis Of Tissue Structure and Dynamics. J. Biomed. Opt..

[97] Oliveira L.M., Carvalho M.I., Nogueira E.M., Tuchin V.V. (2015). Errata: Diffusion characteristics of ethylene glycol in skeletal muscle. J. Biomed. Opt..

[98] Carvalho S., Gueiral N., Nogueira E., Henrique R., Oliveira L., Tuchin V.V. (2017). Glucose diffusion in colorectal mucosa—a comparative study between normal and cancer tissues. J. Biomed. Opt..

[99] Hirshburg J.M., Ravikumar K.M., Hwang W., Yeh A.T. (2010). Molecular basis for optical clearing of collagenous tissues. J. Biomed. Opt..

[100] Weny X., Maoy Z., Han Z., Tuchin V.V., Zhu D. (2010). In vivo skin optical clearing by glycerol solutions: Mechanism. J. Biophotonics.

[101] Matousek P. (2007). Raman signal enhancement in deep spectroscopy of turbid media. Appl. Spectrosc..

[102] Menyaev Y.A., Nedosekin D.A., Sarimollaoglu M., Juratli M.A., Galanzha E.I., Tuchin V.V., Zharov V.P. (2013). Optical clearing in photoacoustic flow cytometry. Biomed. Opt. Express.

[103] Sun Q. (2009). The Raman OH stretching bands of liquid water. Vib. Spectrosc..

[104] Vyumvuhore R., Tfayli A., Duplan H., Delalleau A., Manfait M., Baiilet-Guffroy A. (2013). Effects of atmospheric relative humidity on Stratum Corneum structure at the molecular level: Ex vivo Raman spectroscopy analysis. Analyst.

[105] Choe C., Lademann J., Darvin M.E. (2016). Depth profiles of hydrogen bound water molecule types and their relation to lipid and protein interaction in the human stratum corneum: In vivo. Analyst.

[106] Mudalige A., Pemberton J.E. (2007). Raman spectroscopy of glycerol/D2O solutions. Vib. Spectrosc..

[107] Utz S.R., Tuchin V.V., Galkina E.M. (2015). The dynamics of some human skin biophysical parameters in the process of optical clearing after hyperosmotic solutions topical application. Vestn. Dermatol. Venerol..

[108] Choe C., Choe S., Schleusener J., Lademann J., Darvin M.E. (2019). Modified normalization method in in vivo stratum corneum analysis using confocal Raman microscopy to compensate nonhomogeneous distribution of keratin. J. Raman Spectrosc..

[109] Schulz B., Chan D., Bäckström J., Rübhausen M. (2004). Spectroscopic ellipsometry on biological materials-Investigation of hydration dynamics and structural properties. Thin Solid Films.

[110] Choe C., Schleusener J., Choe S., Ri J., Lademann J., Darvin M.E. (2020). Stratum corneum occlusion induces water transformation towards lower bonding state: A molecular level in vivo study by confocal Raman microspectroscopy. Int. J. Cosmet. Sci..

[111] Nguyen T.T., Happillon T., Feru J., Brassart-Passco S., Angiboust J.F., Manfait M., Piot O. (2013). Raman Comparison of Skin Dermis of Different Ages: Focus on Spectral Markers of Collagen Hydration. J. Raman Spectrosc..

