# Supplementary material for: Confocal Raman Micro-Spectroscopy for Discrimination of Glycerol Diffusivity in Ex Vivo Porcine Dura Mater
Source: Life (Basel). 2022 Oct 1;12(10):1534. doi: 10.3390/life12101534 (PMC9605590; doi:10.3390/life12101534)
Supplement: Supplementary file 1 [file life-12-01534-s001.zip › life-1874356-supplementary.pdf]

life

Supplementary Materials for Confocal Raman Micro-Spectroscopy for Discrimination of Glycerol Diffusivity in Ex Vivo Porcine *Dura Mater*

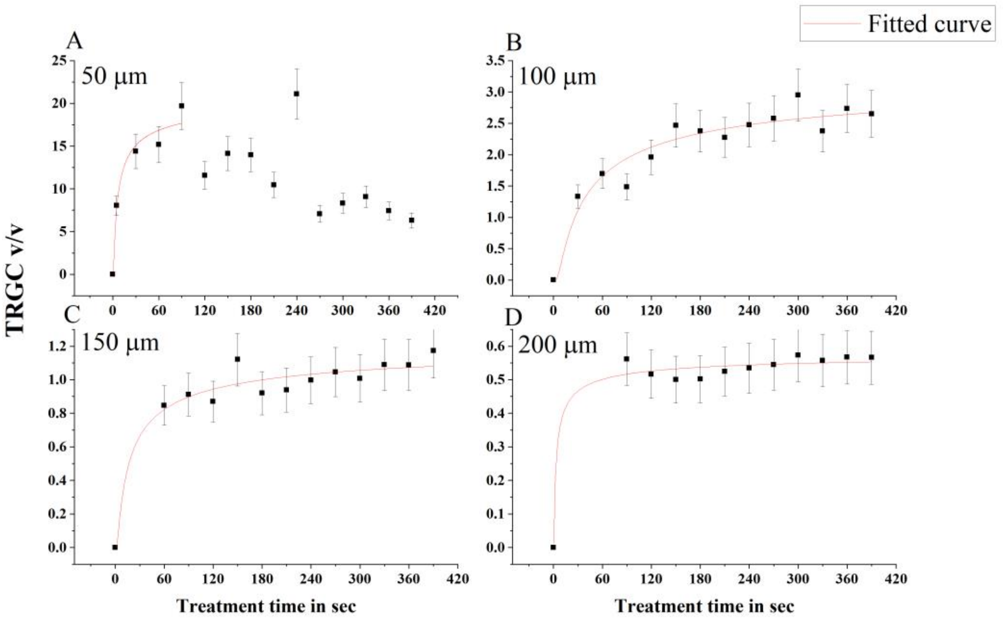

Figure S1. Change of TRGC with treatment time at different depths of 50 μm (**A**), 100 μm (**B**), 150 μm (**C**), and 200 μm (**D**), for porcine DM samples at 938 cm^{-1} impregnated by 50% aqueous glycerol solution.

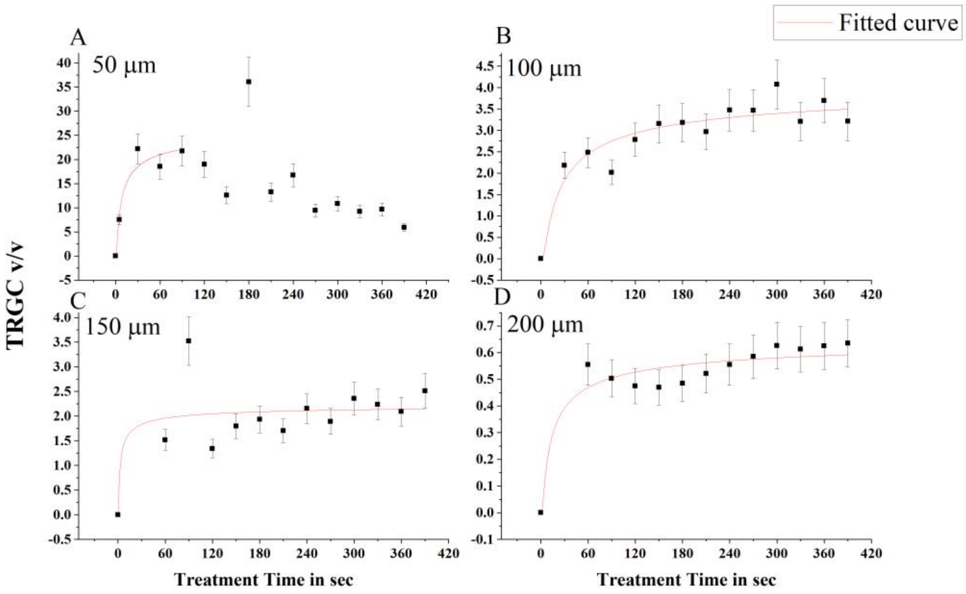

Figure S2. Change of the TRGC with treatment time at different depths of 50 μm (**A**), 100 μm (**B**), 150 μm (**C**), and 200 μm (**D**), for porcine DM samples at 1003 cm^{-1} impregnated by 50% aqueous glycerol solution.

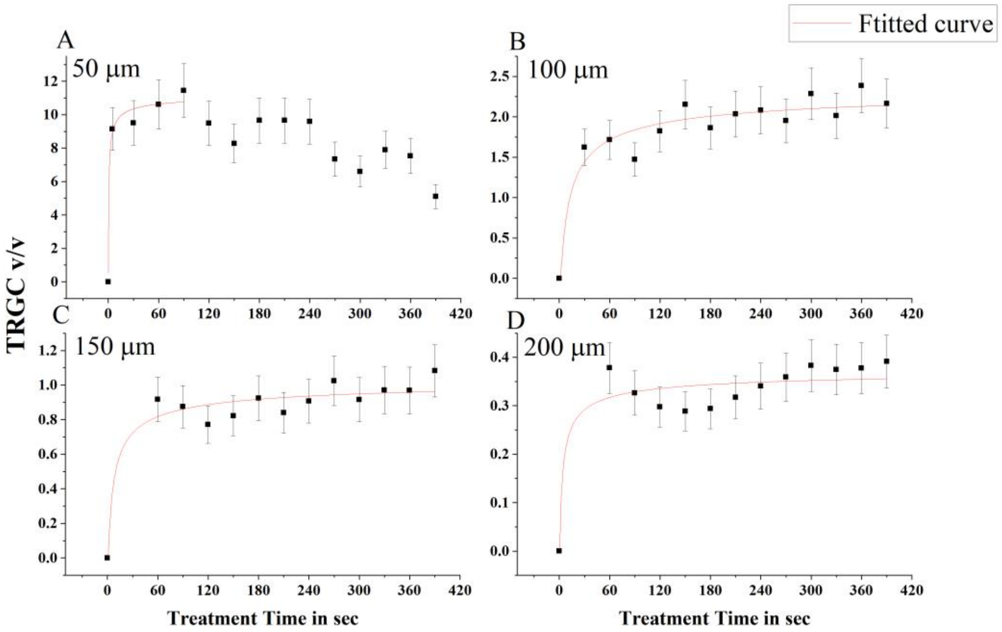

Figure S3. Change of TRGC with treatment time at different depths of 50 µm (**A**), 100 µm (**B**), 150 µm (**C**), and 200 µm (**D**), for porcine DM samples at 1247 cm^{-1} impregnated by 50% aqueous glycerol solution.

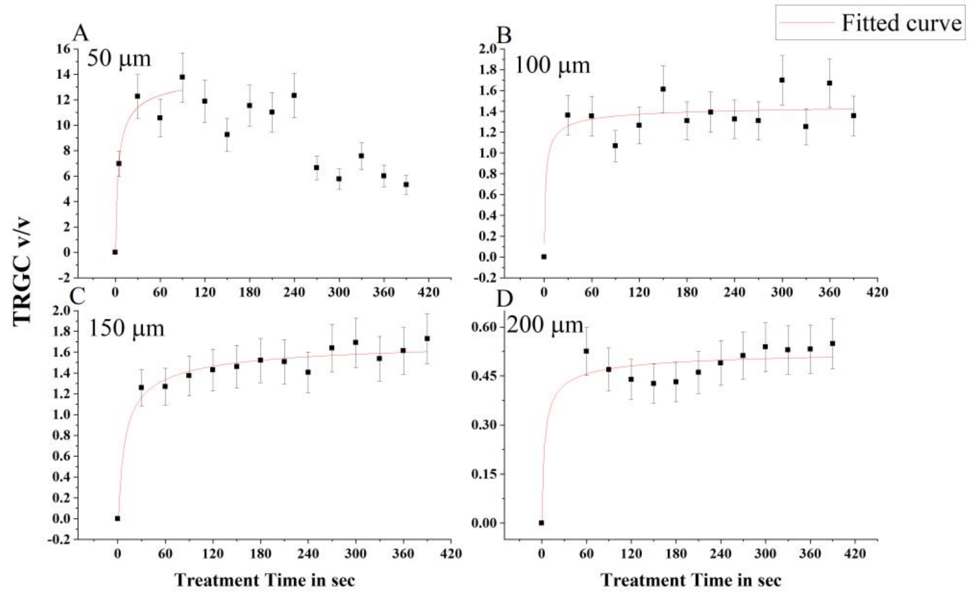

Figure S4. Change of TRGC with treatment time at different depths of 50 µm (**A**), 100 µm (**B**), 150 µm (**C**), and 200 µm (**D**), for porcine DM samples at 1270 cm^{-1} impregnated by 50% aqueous glycerol solution.

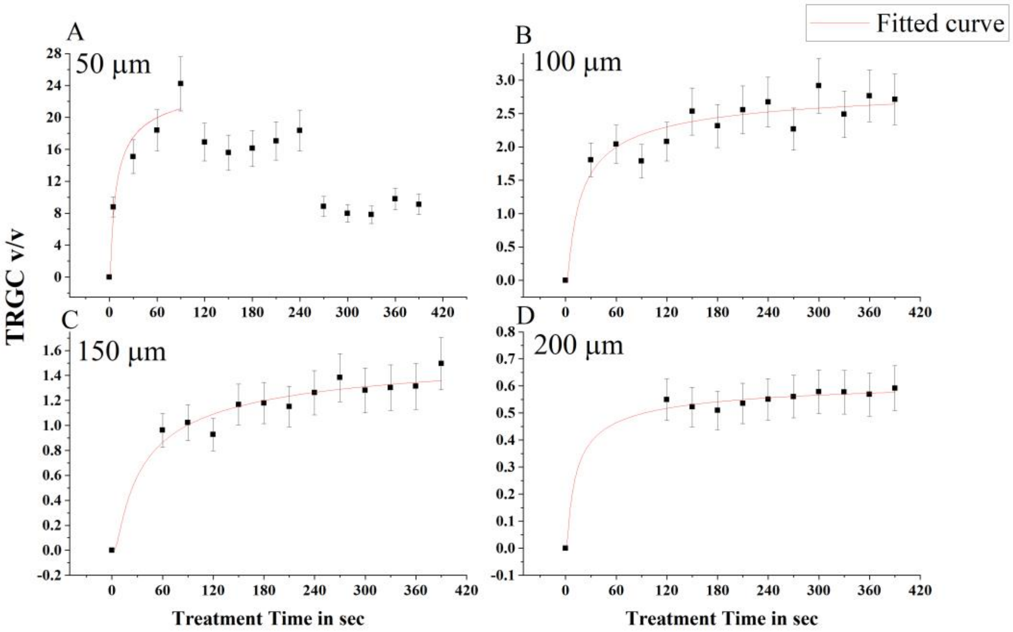

Figure S5. Change of TRGC with treatment time at different depths of 50 μm (**A**), 100 μm (**B**), 150 μm (**C**), and 200 μm (**D**), for porcine DM samples at 1665 cm⁻¹ impregnated by 50% aqueous glycerol solution.

We use Equation (2) for the diffusion coefficient described in[1–3], where C_0 is the concentration of the agent under saturation conditions inside the tissue at depth *x*, *D* a is the diffusion coefficient of the agent. If we stay at a depth of 50 μm, i.e., $x = 50$ μm, then at $t = 0$, $C(t = 0) = 0$ and $C(t = \infty) = C_0$. A complex dependence with saturation and decrease over a short distance may be associated with some artifacts or complex behavior at the liquid-tissue interface. The decrease in the intensity of protein bands over time can be associated with the replacement of the water molecular shell of collagen with glycerol molecules, and each glycerol molecule binding approx. 6 water molecules. Since the concentration of glycerol is high in the surface layers of the tissue, its effect can be significant.

The Raman bands in the interval of frequencies from 1600 to 1700 cm^{-1} usually shows as a triplet bands, Raman band of predominance of proteins with high α-helix content at 1666 cm^{-1}, Raman band at 1635 cm^{-1} is contributions from both α-helix and β-sheet, which is difficult to interpret, and Raman band at 1685 cm^{-1} can be assigned to non-hydrogen bonded. For this reason, we treating as one band due to its dominance in this interval.[4] On other hand, the Raman band at 922 cm^{-1} assigned to the stretching vibration of C–C bond (νC–C) specific of proline residue ring, for this peak, no variation in intensity as function of hydration. In contrast, the Raman band at 938 cm^{-1} correspond to stretching vibration of skeletal C–C, the intensity increased with the hydration. For this reason, we chose this band.[5]

Table S1. Results of the fitting data of the Figures S1, S2, S3, S4 and S5 for DM collagen at different depth and treatment times using the passive diffusion model.

**Depth μm (938 cm^{-1})**	**Time (sec)**	**C_0**	***D* (cm^2/sec)**	**R^2**
**50**	90	21	3.0×10^{-6}	0.967
**100**	390	3.4	1.7×10^{-6}	0.926
**150**	390 starts with 60	1.2	9.4×10^{-6}	0.949
**200**	390 starts with 90	0.5	9.8×10^{-5}	0.978
**Depth μm (1003 cm^{-1})**	**Time (sec)**	***C_0***	***D* (cm^2/sec)**	**R^2**
**50**	90	27	2.5×10^{-6}	0.947
**100**	390	4.2	2.7×10^{-6}	0.886
**150**	390 starts from 60	2.3	6.5×10^{-5}	0.537
**200**	390 starts from 90	0.8	6.2×10^{-6}	0.963

**Depth μm (1247 cm^{-1})**	**Time (sec)**	C_0	D **(cm²/sec)**	**R²**
**50**	90	11	3.5×10^{-5}	0.986
**100**	390	2.4	5.9×10^{-6}	0.918
**150**	390 starts from 60	1.1	2.2×10^{-5}	0.928
**200**	390 starts from 90	0.5	8.1×10^{-6}	0.949
**Depth μm (1270 cm^{-1})**	**Time (sec)**	C_0	D **(cm²/sec)**	**R²**
**50**	90	15	5.0×10^{-6}	0.959
**100**	390	1.5	4.6×10^{-5}	0.835
**150**	390 starts from 60	1.9	9.6×10^{-6}	0.976
**200**	390 starts from 90	0.6	1.1×10^{-5}	0.964
**Depth μm (1665 cm^{-1})**	**Time (sec)**	C_0	D **(cm²/sec)**	**R²**
**50**	90	26	2.3×10^{-6}	0.943
**100**	390	3.1	4.3×10^{-6}	0.919
**150**	390 starts from 60	1.7	4.2×10^{-6}	0.955
**200**	390 starts from 120	0.6	2.5×10^{-5}	0.990

As can be seen from Figure S6, Despite of the partial overlap of the Raman spectrum of the *dura mater* (DM) and 50%-glycerol at specific Raman region. It is possible to recognise that partial overlap of the Raman bands are minimal and could be resolved, which are suitable to study the change in the Raman bands intensity in the DM. However, the obtained results can be potentially underestimated, as 50%-glycerol are partial overlap, where DM has protein-related Raman bands at 938, 1248 and 1270 cm^{-1}. Nevertheless, this underestimation is more pronounced in the superficial depth, where the glycerol has maximum concentration. In deeper DM layers, where the glycerol concentration exists in much lower concentrations, it can be assumed that the contribution from the glycerol-related Raman band is significantly less compared to the protein-related band of the DM, thus substantially reducing the underestimation.

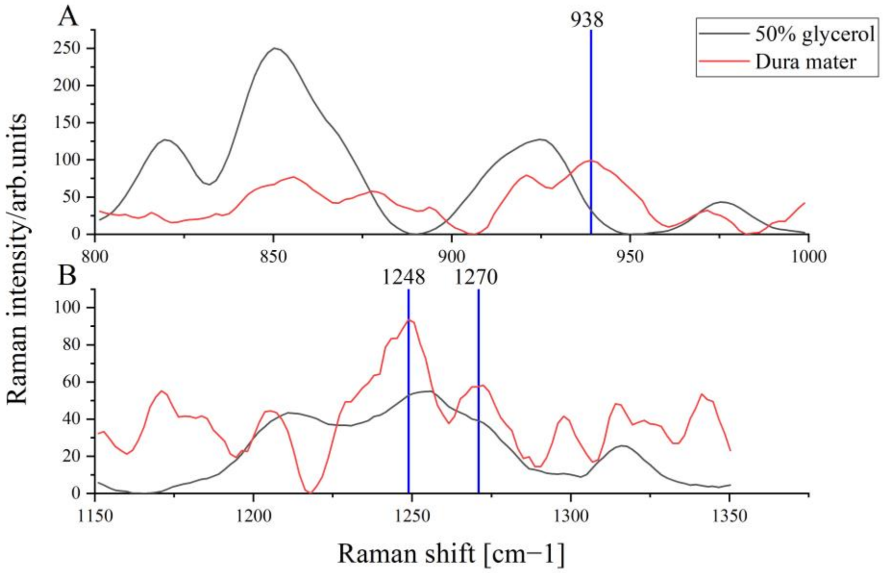

Figure S6. Specific region of FP Raman spectra of control porcine DM at 50 μm depth (red) and 50%-glycerol (black) to recognise the areas where the overlap of the protein-related DM Raman bands at 938, 1248 and 1270 cm^{-1} are minimal.

Reference

1. Bashkatov, A.N.; Tuchin, V. V.; Genina, E.A.; Stolnitz, M.M.; Zhestkov, D.M.; Altshuler, G.B.; Yaroslavsky, I. V. Monte Carlo Study of Skin Optical Clearing to Enhance Light Penetration in the Tissue. Complex Dyn. Fluctuations Biomed. Photonics IV 2007, 6436, 64360Z, doi:10.1117/12.716874.
2. Zhou, F.; Wang, R.K. Theoretical Model on Optical Clearing of Biological Tissue with Semipermeable Chemical Agents. Complex Dyn. Fluctuations, Chaos, Fractals Biomed. Photonics 2004, 5330, 215, doi:10.1117/12.531499.
3. Liu, P.; Huang, Y.; Guo, Z.; Wang, J.; Zhuang, Z.; Liu, S. Discrimination of Dimethyl Sulphoxide Diffusion Coefficient in the Process of Optical Clearing by Confocal Micro-Raman Spectroscopy. J. Biomed. Opt. 2013, 18, 020507, doi:10.1117/1.jbo.18.2.020507.
4. Pezzotti, G.; Boffelli, M.; Miyamori, D.; Uemura, T.; Marunaka, Y.; Zhu, W.; Ikegaya, H. Raman Spectroscopy of Human Skin: Looking for a Quantitative Algorithm to Reliably Estimate Human Age. J. Biomed. Opt. 2015, 20, 065008, doi:10.1117/1.jbo.20.6.065008.
5. Nguyen, T.T.; Happillon, T.; Feru, J.; Brassart-Passco, S.; Angiboust, J.F.; Manfait, M.; Piot, O. Raman Comparison of Skin Dermis of Different Ages: Focus on Spectral Markers of Collagen Hydration. J. Raman Spectrosc. 2013, 44, 1230–1237, doi:10.1002/jrs.4355.
